# Cinnamic Acid Increased the Incidence of *Fusarium* Wilt by Increasing the Pathogenicity of *Fusarium oxysporum* and Reducing the Physiological and Biochemical Resistance of Faba Bean, Which Was Alleviated by Intercropping With Wheat

**DOI:** 10.3389/fpls.2020.608389

**Published:** 2020-12-14

**Authors:** Yuting Guo, J. Lv, Q. Zhao, Yan Dong, K. Dong

**Affiliations:** ^1^College of Resources and Environment, Yunnan Agricultural University, Kunming, China; ^2^College of Animal Science and Technology, Yunnan Agricultural University, Kunming, China

**Keywords:** intercropping, physiological resistance, *Fusarium* wilt, *Fusarium oxysporum*, faba beans, cinnamic acid

## Abstract

**Background:**

Continuous cropping has resulted in the accumulation of self-toxic substances in faba beans which has restricted their global production. Intercropping is widely used to alleviate these problems.

**Aims:**

To explore the role of cinnamic acid stress in faba bean physiology and disease resistance, and the potential mitigating effects of intercropping the faba bean with wheat.

**Methods:**

Faba bean seedlings were grown with or without wheat in both field and hydroponic conditions in the presence of different cinnamic acid concentrations and *Fusarium oxysporum* (FOF), the occurrence of. *Fusarium*-mediated wilt and oxidative stress, as well as plant growth indices and the anti-pathogen defense system were analyzed.

**Results:**

Cinnamic acid significantly increased *Fusarium* pathogenicity, inhibited the activity of defense enzymes and reduced the ability of plants to resist pathogens, indicating the importance of cinnamic acid in the promotion of *Fusarium* wilt resulting in reduced seedling growth. Intercropping with wheat improved plant resistance by alleviating cinnamic acid-induced stress, which promoted crop growth and decreased the incidence and disease index of *Fusarium* wilt.

**Conclusion:**

Cinnamic acid promotes *Fusarium* wilt by stimulating pathogen enzyme production and destroying the defense capability of faba bean roots. Intercropping reduces *Fusarium* wilt by alleviating the damage caused by cinnamic acid to the defense system of the faba bean root system.

## Introduction

The continuous planting and harvesting of single crops is a common practice in modern agriculture (Young, 1984; Grodzinsky, 1992), which has resulted in restricted growth and decreased crop yields (Huang et al., 2013) by increasing the risk of soil-borne disease (Asao et al., 2004; Ren et al., 2008; Wu et al., 2009). Studies have shown that there is a significant correlation between the accumulation of autotoxic substances in the rhizosphere and the occurrence of soil-borne diseases (Ye et al., 2006; Wu et al., 2008b). Phenolic acids are aromatic secondary metabolites and that are typical autotoxic substances found in plant root exudates. Cinnamic acid is a well-documented toxic phenolic acid. Studies have shown that cinnamic acid can significantly affect the physiological, biochemical, and defense responses of plants, and can increase the risk of plant infection with *Fusarium* wilt (Huang et al., 2013). Ye et al. (2006) found that increased concentrations of cinnamic acid increase the activity of SOD GPX APX CAT, cell membrane peroxidation, and ion leakage in cucumber plants, leading to oxidative damage and increasing the risk of cucumber infection with *Fusarium* wilt. On the other hand, phenolic acids can also cooperate with soil-borne pathogens to further increase the pathogen’s infective ability and cause plant diseases. Wu et al. (2008a) observed that exogenous cinnamic acid not only has a strong inhibitory effect on the biomass of FON but also strongly stimulates FON production of mycotoxins and enzymes related to pathogenesis, reaching 27–2,630%. Wu et al. (2015) reported similar results in the Lanzhou lily. Therefore, reducing the toxic effects of phenolic acid in root exudates may be the key to alleviating the obstacles associated with continuous cropping in plants.

Several chemical and biological methods have been developed to control plant diseases. However, chemical preparations have obvious side-effects, such as causing disease-resistance in pathogens and environmental pollution (Minuto et al., 2006). In addition, biopharmaceuticals may become unstable in field conditions. *Pythium oligandrum* has attracted much attention as a biological control agent, but under field conditions, the effects of this biological control agent are drastically reduced because it cannot survive in plants (Gerbore et al., 2014). Intercropping is an agricultural practice in which two or more crops are planted in close proximity, allowing biodiversity to improve crop resistance and yield. Scholars have found that after selecting the correct companion crops, intercropping can significantly control crop diseases and improve resource utilization efficiency (Li et al., 2013). For example, Guo et al. (2020) and Xiao et al. (2018) found that faba bean/wheat intercropping significantly controlled chocolate spot in the faba bean, promoted the efficient use of N, and reduced the application of N fertilizer. Gao et al. (2014) and Yang et al. (2014) found in the corn/soybean intercropping system that the direct interaction of soybean and corn root system can inhibit the growth and reproduction of pathogens, while at the same time increasing the expression of host plant PR genes and their corresponding enzyme activities, and increasing the soybean’s resistance to red crown rot. However, these studies did not analyze the physiological effects of intercropping.

Faba beans are grown all over the world as food and animal feed (Alghamdi et al., 2012). However, continuous cropping for many years has led to the frequent occurrence of soil-borne diseases, among which fusarium wilt is the most serious (Stoddard et al., 2010). After the faba bean was infected with *Fusarium* wilt, the plant began to lose water, withered, and finally died. This has affected the production of faba beans worldwide and restricted their sustainable development. *Fusarium* wilt is caused by *Fusarium oxysporum*, which can survive for long periods in the soil, even in the absence of faba beans, and is, thus, difficult to control (Stoddard et al., 2010). In Yunnan and southwestern China, faba beans are usually planted together with wheat to improve the efficient use of nutrient resources and control faba bean diseases. Our previous research found that the intercropping of faba beans and gramineous plants can increase the diversity of root microbes, thereby effectively preventing fusarium wilt. In this study, we detected high levels of cinnamic acid in the roots of monocultured faba beans, which was alleviated when grown with wheat. Therefore, we hypothesized that cinnamic acid can aggravate *Fusarium* wilt as a self-toxic substance, and analyzed its effect on the growth and pathogenicity of *Fusarium oxysporum* (FOF), along with the mitigating influences of intercropping.

## Materials and Methods

### Test Materials

The 89-147 faba bean (*Vicia faba* L.) and Yunmai53 wheat (*Triticum aestivum* L.) varieties were obtained from the Yunnan Academy of Agricultural Sciences (Yunnan Sheng, China). Cinnamic acid was bought from China Pharmaceutical Group Shanghai Medical Instrument Co. Ltd. (Shanghai, China). FOF was isolated from a faba bean monoculture at the Plant–Microbe Laboratory at Yunnan Agricultural University, cultured in potato dextrose agar (PDA) for 7 days at 28°C, and the mycelia were stored at 4°C. Galacturonic acid, pectin, and cellulose were bought from Ryon Co. (Shanghai, China), Sigma Co. (St. Louis, MO, United States), and Tokyo Chemical Industry (Tokyo, Japan) respectively.

### Field Trials

The field test was conducted in Efeng village (24°11’n, 102°24’e, 1540 m above sea level), Eshan county, Yuxi, South Yunnan province from October 2016 to May 2017 and October 2017 to May 2018. It lies in the humid subtropical zone and has paddy soil type with the topsoil (0∼20 cm) containing 28.5 g kg^–1^ organic matter, 2.3 g kg^–1^ total nitrogen, 0.71 g kg^–1^ total phosphorus, 17.6 g kg^–1^ total potassium, 106 mg kg^–1^ alkali-hydrolyzed nitrogen, 33.5 mg kg^–1^ available phosphorus, and 98.5 mg kg^–1^ available potassium, pH 6.7.

**FIGURE 1:**
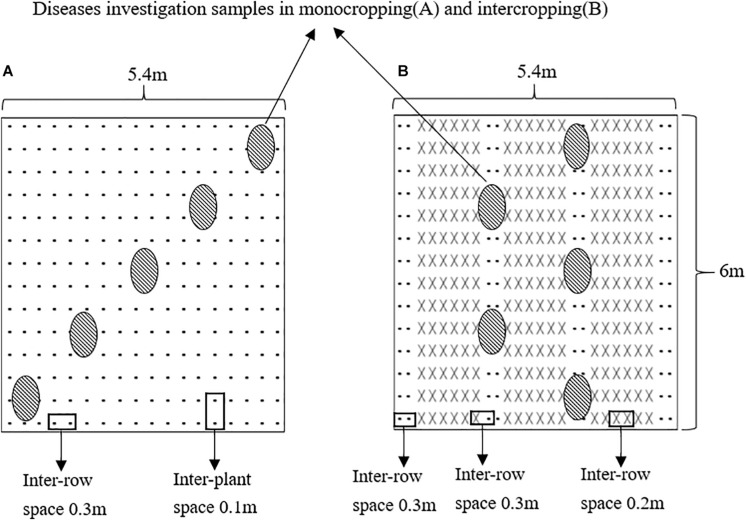
Diagram of the planting patterns in the field experiments: **(A)** the monocropping faba bean plot, **(B)** the intercropping faba bean with wheat plot (-represents faba bean and × represents wheat).

The faba beans were monocropped (MF) or intercropped with wheat (IF) in plots measuring 5.4 m × 6 m with an area of 32.4 m^2^. As shown in Figure 1, the MF faba bean plants were sown at 0.1 m spacing in six rows, and the row spacing was 0.3 m. In the IF plot, six rows of wheat and two rows of faba beans were planted alternately for a total of three and four strips, respectively. The spacing between the faba bean rows and the faba bean and wheat rows was 0.3 m, whereas that between the wheat rows was 0.2 m. The faba bean plants from the outermost rows of the first and fourth strips were not sampled. In addition, a 1 m-wide faba bean strip was planted around the entire test field as a protection line. Each treatment was repeated 6 times in 12 random blocks. No pesticides, fungicides, or herbicides were applied throughout the growth period. Other management was carried out according to the local agronomic customs.

### Greenhouse Cultivation

We set up hydroponic experiments in the greenhouse. The faba beans were grown in pots in a greenhouse at Yunnan Agricultural University in the MF (six faba bean plants per pot) or IF (three faba bean plants and nine wheat plants each per pot) mode from October 2016 to May 2017 and October 2017 to May 2018. The plants were grown under illumination for 14 h daily at 26°C/22°C in the presence of 0, 50, 100, and 200 mg L^–1^ cinnamic acid. The experiment was repeated thrice.

Faba bean seeds were soaked at room temperature for 24 h, and germinated in sterile quartz sand at 25°C. Once the seedlings grew 4–6 leaves about 10 cm high, they were transplanted into 3 L plastic pots (25 cm in upper diameter, 13 cm in lower diameter and 16 cm in height), which contains 2 L of Hoagland nutrient solution of different concentrations of cinnamic acid, and grown with or without the wheat seedlings. In the following time we replaced the Hoagland nutrient solution with the same cinnamic acid concentration and the 1 × 106 ml^–1^ FOF spore suspension every 3 days, and the position of the pots was changed randomly. The nutrient solution was kept in a pot and ventilated with a ventilator pump 24 h a day.

### Evaluation of *Fusarium* Wilt

We evaluated the potted faba beans for *Fusarium* wilt 40 days after FOF inoculation, selecting three faba beans from each pot for disease assessment. We also evaluated the faba beans in the field 60 days after sowing. In the MF plot, five diagonal points were randomly selected and three plants from each point were analyzed (15 plants for each plot). In the IF plot, five points were selected on the two faba bean belts (two points in the first belt and three points in the second belt), and three plants were surveyed at each point (15 plants per plot) (Figure 1). The severity of disease was scored at different stages as: 0 – no symptoms of infection, 1 – slight plaques or discoloration at the base of the stem or peripheral roots, 2 – uneven lesions at the base of the root or stem, 3 – uniform lesions, discoloration or wilting in 1/3 to 1/2 of the stem base or root and reduction in lateral roots, 4 – completely discolored or withered roots or stem base, 5 – complete wilting of the plant and death. The disease index and wilt incidence were calculated as:

$$\text{Incidence}=\frac{\mathrm{Number}\,\mathrm{of}\,\mathrm{diseased}\,\mathrm{plants}}{\mathrm{total}\,\mathrm{number}\,\mathrm{of}\,\mathrm{plants}\,\mathrm{investigated}}\times 100\%$$

(1) $$\mathrm{Disease}\,\mathrm{index}=\frac{\sum \left(\mathrm{Number}\,\mathrm{of}\,\mathrm{diseased}\,\mathrm{plants}\,\mathrm{at}\,\mathrm{each}\,\mathrm{level}\times \mathrm{level}\right)}{\mathrm{The}\,\mathrm{highest}\,\mathrm{level}\times \mathrm{total}\,\mathrm{number}\,\mathrm{of}\,\mathrm{plants}\,\mathrm{investigated}}\times 100$$

### Collection of Rhizosphere Soil

Rhizosphere samples were collected 60 days after sowing. After examining the 15 faba bean plants from each plot as described above, the rhizosphere soils were mixed and stored in an ice-box until further analysis about 2 h.

### Determination of Phenolic Acid Content in Rhizosphere Soil

Thirty grams rhizosphere soil was dispersed in 200 mL extract (pH 5.6, including 200 μmol L^–1^ MgCl_2_, 100 μmol⋅L^–1^ KCl, 600 μmol⋅L^–1^ CaCl_2_, and 5 μmol⋅L^–1^ H_3_BO_3_) and leached for 1 h with periodic shaking. About 20 mL of the solution was aliquoted into a 50 mL centrifuge tube, and microbial activity was inhibited by adding 2–3 drops of 98% phosphoric acid. After freeze-drying, the powder was resuspended in 1 mL water and passed through a 0.45 μm membrane. The phenolic acid content was measured by high-performance liquid chromatography (Agilent 1260 Infinity, Agilent Technologies, Santa Clara, CA, United States) using vanillic acid, *p*-hydroxybenzoic acid, syringic acid, salicylic acid, ferulic acid, benzoic acid, and cinnamic acid (chromatographically pure) as standards. A Kinetex core-shell chromatography column measuring 2.6 μm × 4.6 × 100 mm was used with the following parameters: column temperature – 30°C, injection volume – 10 μL, DAD detector – 280 nm, and flow rate – 0.5 mL min^–1^. The mobile phase A was methanol (chromatographic grade) and B was 0.1% phosphoric acid-water. The following gradient was used for elution: mobile phase B 80% (0 min) → 5% (15 min) → 5% (18 min) → 80% (18.5 min) → 0% (20 min) → stop (25 min). Phenolic acid types were determined by retention time and the content of each phenolic acid was calculated by the external standard method.

### Evaluation of FOF Growth and Conidial Germination

Mycelial discs measuring 9 mm in diameter were plated onto PDA and cultivated for 7 days at 28°C. The colony diameter was measured radially in three directions on days 3 and 7. A 9 mm agar plug was cut from the 7-day-old culture and inoculated into 15 ml PD medium containing 0, 50, 100, or 200 mg/L cinnamic acid, and incubated for 7 days at 28°C with constant shaking at 170 rpm. The culture broth was filtered and dried at 80°C for 12 h and weighed to determine the fungal biomass. To determine spore germination, the 7-day-old mycelia on PDA were washed with sterile water, and the spores were collected by filtering through four layers of gauze. The spore suspension was diluted to ≤1 × 103 CFU/ml, and 0.1 ml of spores were plated on each 2% (w/v) water agar plate containing 0, 2.5, 5, 10, 20, 40, 80, or 160 mg/L cinnamic acid. The plates were incubated for 3 days at 28°C and the number of colonies were counted. Each experiment was repeated thrice.

### Extraction and Quantification of Mycotoxin

The 7-day-old mycelia were inoculated into 125 ml Richard’s medium (Gaumann, 1957; 5 g KH_2_PO_4_, 0.02 g FeSO_4_, 10 g KNO_3_, 34 g glucose, and 2.5 g MgSO_4_ in 1L distilled water) in a 250 ml Erlenmeyer flask and cultured at 28°C for 15 days with constant shaking at 180 rpm. The broth was centrifuged for 10 min at 5,000 rpm, and the supernatant was filtered through a 0.45 μm mesh to remove the mycelia and spores. The filtrate was then hot-pressed to obtain the crude toxin, which was then mixed with the same amount of ethyl acetate, shaken for 2 min, and left undisturbed for 30 min. The organic phase was aspirated and centrifuged for 10 min at 4,000 rpm, and the supernatant was dried at 40°C. After dissolving in 5 ml ethyl acetate, the absorbance was measured at 269 nm.

### Evaluation of FOF Hydrolase Activity

The crude enzyme solution was prepared using 1% induction substrate (pectin, cellulose) in synthetic medium. A 7-day old 9-mm mycelial disc was inoculated into 25 ml of the above medium in a 100 ml flask and cultured at 28°C for 7 days with constant shaking at 200 rpm. The culture broth was centrifuged at 1,789 *g* for 10 min, and the supernatant was filtered through 0.45 μm membrane. The resulting crude enzyme filtrate was stored at 4°C.

To prepare the DNS reagent, 3.15 g of 3,5-dinitrosalicylic acid (chemically pure) was dissolved in 500 ml water at 45°C, and 100 ml 0.2 g/ml sodium hydroxide was added till the solution was clear. After addition of 91 g potassium sodium tartrate, 2.5 g phenol, and 2.5 g anhydrous sodium sulfite, the solution was diluted with 300 ml water and heated at 45°C with constant stirring until the chemicals were completely dissolved. The solution was cooled to room temperature, diluted to 1,000 ml, and stored in the dark at room temperature for 7 days.

Pectinase activity was determined as previously described (Cao et al., 2011). Briefly, 1.9 ml 1% pectin (in 0.1 mol L^–1^ acetate buffer, pH = 4.6) was incubated with 0.1 ml crude enzyme solution at 40°C in a water bath for 30 min, followed by addition of 1.5 ml DNS reagent. The solution was boiled for 5 min, cooled, and diluted to 30 ml with distilled water. The absorbance was measured at 500 nm. One enzyme activity unit was defined as the amount of enzyme required to produce 1 μmol of galacturonic acid per minute.

Cellulase activity was determined based on the method of Cao et al. (2011). The substrate was prepared by dissolving 0.1 g sodium nitrate in 10 ml of 50 mmol⋅L^–1^ citric acid-sodium citrate buffer, pH 5. One milliliter each of the crude enzyme solution, citric acid-sodium citrate buffer, and the substrate were incubated at 50°C for 30 min. Colorimetric measurement at 540 nm was performed as described above. One enzyme activity unit was defined as the amount of enzyme required to produce 1 μmol of glucose per minute. All experiments were performed thrice.

### Evaluation of Oxidative Stress Levels

POD activity was measured as described previously (Muñoz-Muñoz et al., 2009) (Quintanilla-Guerrero et al., 2008). Briefly, 1 g root samples were ground and the homogenate was mixed with the appropriate amount of phosphate buffer. After centrifuging at 3,000 rpm for 10 min, the supernatant was aspirated. In a 25 ml volumetric flask, 0.1 ml of the enzyme was mixed with 1 ml 2% H_2_O_2_, 2.9 ml 0.05M phosphate buffer, and 1 ml 0.05M guainol, and incubated in a 34°C water bath for 3 min. The absorbance at 470 nm was measured every five minutes.

CAT activity was also measured as previously described (Manoranjan and Dinabandhu, 1976; Garcia-Limones et al., 2002). The root homogenate was centrifuged for 15 min at 4,000 rpm, and 2.5 ml of the supernatant and 0.1M H_2_O_2_ were mixed and incubated at 30°C in a water bath for 10 min. After adding 2.5 ml 10% H_2_SO_4_, the solution was titrated with 0.1M KMnO_4_ until the solution turned pink. One CAT activity unit is expressed as the number of milligrams of H_2_O_2_ decomposed in 1 minute per gram of fresh weight sample (mg g^–1^ min^–1^).

To measure the content of malondialdehyde (MDA), the end-product of membrane lipid peroxidation (Bird, 1983), 0.5 g plant sample was homogenized in 5 ml of 5% TCA, and centrifuged at 3,000 rpm for 10 min. The supernatant was aspirated, and 2 ml was boiled with the same volume of 0.67% TBA for 30 min, cooled, and centrifuged. The absorbance was measured at 450, 532, and 600 nm.

### Evaluation of Chitinase and β-1, 3-Glucanase Activity

Chitinase activity was measured using a commercially available kit (Nanjing Jiancheng Bioengineering Research, China). One unit of chitinase activity is defined as the mass of chitin that breaks down into 1 mg of n-acetyl-d-(+)-glucosamine per gram of tissue per hour. The β-1,3-glucanase activity was measured using a kit from Beijing Sulei Technology Co. Ltd. (Beijing, China), and unit activity was defined as 1 mg of reducing sugar produced per gram of tissue per hour.

### Statistical Analysis

SPSS (ver. 20.0; IBM Corp., Armonk, NY, United States) and Microsoft Excel 2010 (Microsoft Corp., Redmond, WA, United States) were used for statistical analysis. Significant differences between treatments were evaluated using 2-factor ANOVA, followed by Duncan’ test at the 5% probability level.

## Results

### Intercropping Controlled *Fusarium* Wilt in Faba Beans

**FIGURE 2:**
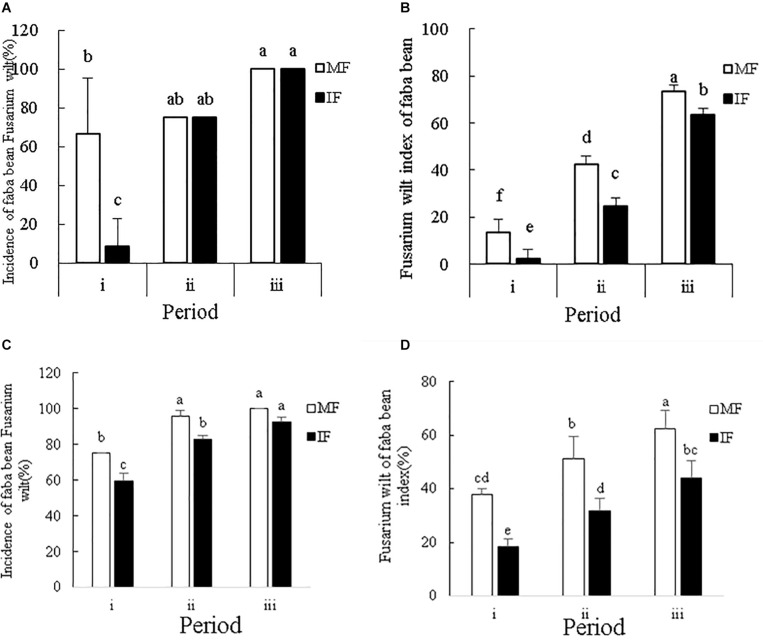
**(A)** Incidence and **(B)** disease index of *Fusarium* wilt in faba beans at different stages of growth from October 2016 to May 2017. **(C)** Incidence and **(D)** disease index of *Fusarium* wilt in faba beans at different stages of growth from October 2017 to May 2018. Data represent mean ± standard error of six biological replicates. i – flowering period, ii – fruiting period and iii – mature period. MF: Faba bean monocropping; IF: wheat-faba bean intercropping. Different letters for each index indicate significant differences at *p* < 0.05 level.

In the 2016–2017 field experiment, we found that the incidence and disease index of wilt in faba bean plants were highest in the mature stage, followed by the fruiting/pod and flowering stages, in that order (Figures 2A,B). Intercropping with wheat significantly decreased the wilt incidence rate and disease index at the flowering stage of faba bean plants by 87.5 and 83.35%, respectively. In the pod and mature stages, however, intercropping only reduced the disease index (42.11% in the pod stage) without any significant effect on the incidence of *Fusarium* wilt.

In the 2017–2018 field experiment, we found that the incidence and disease index of wilt in faba bean plants was highest in the mature stage, followed by the fruiting/pod and flowering stages, in that order (Figures 2C,D). In the flowering and fruiting stages, intercropping with wheat significantly decreased the wilt incidence rate, but this effect was not seen in the mature period. In all stages, intercropping with wheat significantly decreased the wilt index. Taken together, intercropping faba beans with wheat can effectively decreased disease susceptibility in the early stages of growth.

### Intercropping Decreased the Phenolic Acid Content in the Rhizosphere of Faba Beans

**FIGURE 3:**
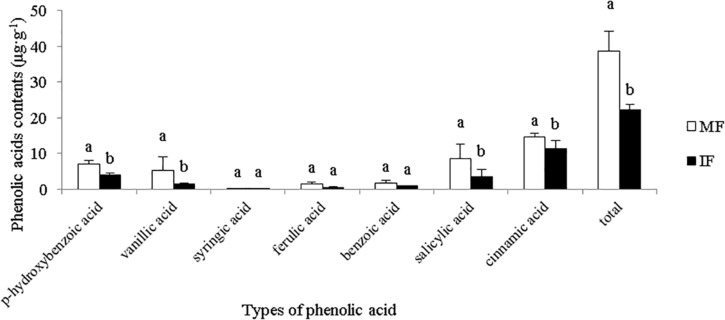
Contents of different phenolic acids in the rhizosphere of field-cultivated plants. Data represent mean ± standard error of six biological replicates. Different letters on the same phenolic acid indicate significant differences under different planting modes at *p* < 0.05 level.

As shown in Figure 3, we detected seven phenolic acids in the rhizosphere of faba beans, of which cinnamic acid was predominant. The content of cinnamic acid was the most, the content of cinnamic acid in the monocropped plants accounted for 37.56% of the total phenolic acid. Compared with monocropping, intercropping significantly reduced the content of cinnamic acid in the rhizosphere. These results suggest that cinnamic acid may play an important role in the occurrence of faba wilt.

### Cinnamic Acid Inhibited FOF Growth and Sporulation but Enhanced FOF Pathogenicity

**FIGURE 4:**
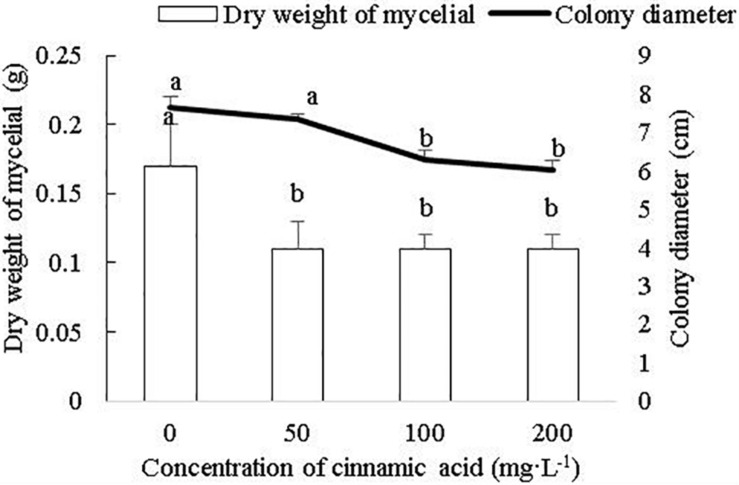
Effects of cinnamic acid on FOF growth and biomass. Data represent mean ± standard error of three biological replicates. Different letters for each index indicate significant differences at *p* < 0.05 level in the bar chart and line chart, respectively.

**FIGURE 5:**
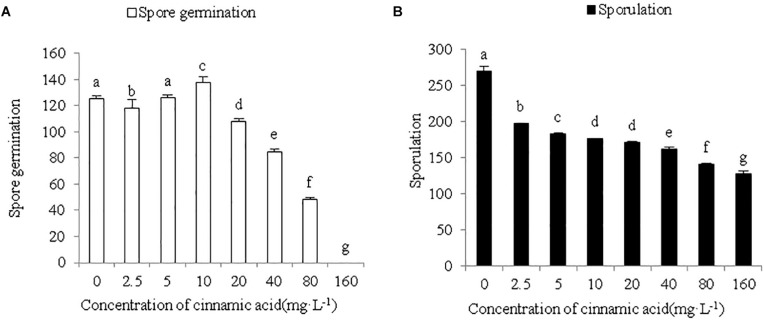
Effects of cinnamic acid on the germination **(A)** and sporulation **(B)** of FOF spores. Data represent mean ± standard error of three biological replicates. Different letters for each index indicate significant differences at *p* < 0.05 level.

To determine whether cinnamic acid increases the susceptibility of faba beans to *Fusarium* wilt, we analyzed its effect on the growth and pathogenicity of *F. oxysporum in vitro*. As shown in Figure 4, FOF growth was significantly inhibited by the addition of cinnamic acid in terms of both mycelial dry weight and colony size. The high dose of 200 mg/L reduced the dry weight of mycelia by 41.18% compared to the control. With the 50 and 100 mg/L dose, there was a decrease in a range similar to the 200 mg/L dose. Compared with the control, cinnamic acid at concentrations of 100 and 200 mg/L significantly inhibited the radial colony growth of the FOF and the inhibitory effect reached the maximum 20.97% at 200 mg/L (Figure 4). Spore germination was reduced with 2.5 mg/L cinnamic acid, increased with10 mg/L, and was later reduced above 20 mg/L. On the other hand, cinnamic acid significantly inhibited the sporulation of FOF in a concentration-dependent manner (Figure 5). Interestingly, 100 and 200 mg/L cinnamic acid increased the cellulase activity of the cultured mycelia by 58.17 and 96.09%, respectively. The secreted amount of fusaric acid also increased by 166.37, 203.93, and 247.17% in the presence of 50, 100, and 200 mg/L cinnamic acid, respectively. Pectinase activity was also slightly improved in the presence of cinnamic acid (Table 1). Taken together, cinnamic acid had an inhibitory effect on the growth and sporulation of FOF but significantly improved the production of pathological substances, suggesting that cinnamic acid is a self-toxic substance for faba beans.

**TABLE 1:** Effect of cinnamic acid on the synthesis of FOF pathological factors.

Concentration (mg L^–1^)	0	50	100	200
Pectinase activity (U ml^–1^ min^–1^)	0.11 ± 0.01*a*	0.13 ± 0.01*b*	0.14 ± 0.00*b*	0.13 ± 0.01*b*
Cellulase activity (mmol min^–1^)	8.94 ± 3.09*a*	13.66 ± 1.47*ab*	14.14 ± 1.64*b*	17.53 ± 2.46*b*
Fusaric acid content (mg L^–1^)	30.57 ± 4.76*a*	81.43 ± 5.08*b*	92.91 ± 2.51*b*	106.13 ± 4.76*c*

*Data represent mean ± standard error of three experiments. Different letters for the same index indicate significant differences at *p* < 0.05 level.*

### Intercropping Alleviated Cinnamic Acid-Induced Risk to *Fusarium* Wilt in Faba Beans

**FIGURE 6:**
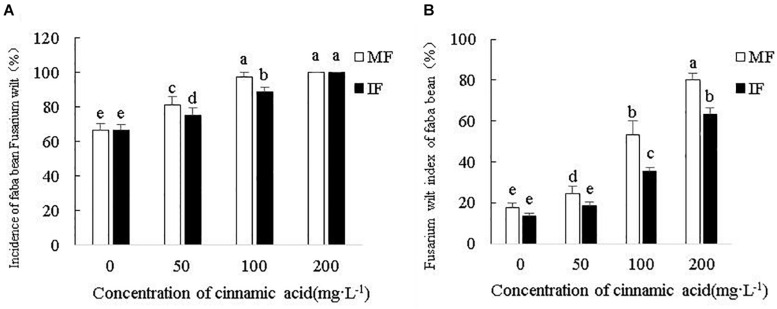
Effects of cinnamic acid on the **(A)** incidence and **(B)** disease index of Fusarium wilt under different cropping systems. Data represent mean ± standard error of three biological replicates. Different letters for each index indicate significant differences at *p* < 0.05 level.

As shown in Figure 6, the exogenous addition of cinnamic acid significantly increased the incidence and disease index of *Fusarium* wilt compared to the control in a concentration-dependent manner. Intercropping with wheat significantly reduced the incidence of *Fusarium* wilt by 7.4 and 9.21% under 50 and 100 mg/L cinnamic acid, respectively, compared to the monocultured faba beans. In contrast, the incidence rate at the high dose of 200 mg/L was unaffected by growing both crops together (Figure 6A). The disease index was significantly decreased upon intercropping by 24.55, 33.32, and 20.81% in the presence of 50, 100, and 200 mg/L cinnamic acid, respectively (Figure 6B). Taken together, intercropping faba beans with wheat can significantly decrease the risk of *Fusarium* wilt under cinnamic acid stress.

### Intercropping Improved the Growth of *Fusarium*-Infected Faba Bean Plants Under Cinnamic Acid Stress

As shown in Table 2, cinnamic acid treatment significantly reduced all legume growth indices, except for the main root length and root-cap ratio. Compared to the untreated control, 50, 100, and 200 mg/L cinnamic acid reduced the number of leaves by 24.64, 40.01, and 45.41%, maximum l, and 46.04%, respectively. In addition, cinnamic acid also decreased the plant height by 15.29, 30.23, and 41.80%, stem dry weight by 25.56, 58.74, and 68.16%, and root dry weight by 27.78, 62.96, and 74.07% at the concentrations of 50, 100, and 200 mg/L, respectively. The higher doses of 100 and 200 mg/L reduced the main root length by 8.51 and 27.82%, respectively, and the root-crown ratio was significantly reduced by 16.67% by 200 mg/L cinnamic acid. As shown in Table 2, these growth indices improved significantly when the faba bean seedlings were intercropped with wheat. In the absence of cinnamic acid, wheat intercropping increased the number of leaves, main root length, and the stem and root dry weight of faba beans by 10.75, 8.18, 47.09, and 59.26%, respectively. Furthermore, the maximum leaf length, width, stem dry weight, and root dry weight of the intercropped faba bean plants increased by 13.24, 14.53, 45.18, and 53.85%, respectively, compared to the monocrops under 50 mg/L cinnamic acid. Likewise, the intercropped plants showed a 16.69, 12.83, 33.7, 50, and 9.09% increase in the number of leaves, maximum leaf length, stem dry weight, root dry weight, and root-shoot ratio, respectively, under 100 mg/L cinnamic acid. At the high dose of 200 mg/L however, wheat intercropping only increased the maximum leaf length (26%) and root-shoot ratio (10%) compared to the monocultured plants. Taken together, wheat and faba bean intercropping can significantly improve the growth indices of the latter under *Fusarium* and cinnamic acid stress.

**TABLE 2:** Effects of cinnamic acid on the growth indices of FOF-inoculated faba bean seedlings under different planting conditions.

Growth parameters	0 mg L^–1^		50 mg L^–1^		100 mg L^–1^		200 mg L^–1^	
	MF	IF	MF	IF	MF	IF	MF	IF
Leaf number per plant	21.67 ± 0.58*b*	24.00 ± 1.00*a*	16.33 ± 2.89*cd*	17.33 ± 0.58*c*	13.00 ± 1.00*e*	15.17 ± 1.04*d*	11.83 ± 0.29*e*	12.67 ± 0.76*e*
Max leaf length/cm	8.73 ± 0.38*a*	9.37 ± 0.12*a*	6.80 ± 0.61*cd*	7.70 ± 0.75*b*	6.47 ± 0.38*d*	7.30 ± 0.20*bc*	4.50 ± 0.10*e*	5.67 ± 0.29*f*
Max leaf width/cm	5.43 ± 0.51*a*	5.53 ± 0.12*a*	4.13 ± 0.12*c*	4.73 ± 0.40*b*	3.77 ± 0.55*cd*	4.03 ± 0.12*c*	2.93 ± 0.15*e*	3.31 ± 0.20*ed*
Height/cm	39.23 ± 0.49*a*	37.87 ± 1.44*a*	33.23 ± 1.48*b*	30.98 ± 0.58*c*	27.33 ± 1.11*d*	23.81 ± 1.39*e*	22.83 ± 0.58*e*	18.67 ± 0.55*f*
Main root length/cm	18.33 ± 1.46*b*	19.83 ± 0.97*a*	17.53 ± 0.42*bc*	18.07 ± 0.21*b*	16.77 ± 0.45*c*	17.47 ± 0.15*bc*	13.23 ± 0.31*e*	13.50 ± 0.29*e*
Shoot dry weight/g	2.23 ± 0.05*b*	3.28 ± 0.08*a*	1.66 ± 0.11*c*	2.41 ± 0.10*b*	0.92 ± 0.21*e*	1.23 ± 0.14*d*	0.71 ± 0.08*f*	0.90 ± 0.04*ef*
Root dry weight/g	0.54 ± 0.03*b*	0.86 ± 0.06*a*	0.39 ± 0.05*a*	0.60 ± 0.04*b*	0.20 ± 0.05*c*	0.30 ± 0.01*e*	0.14 ± 0.04*b*	0.20 ± 0.01*e*
Root-shoot ratio/%	0.24 ± 0.02*ab*	0.26 ± 0.01*a*	0.23 ± 0.02*ab*	0.25 ± 0.01*a*	0.22 ± 0.01*bc*	0.24 ± 0.03*ab*	0.20 ± 0.02*c*	0.22 ± 0.01*bc*

*Data represent mean ± standard error of three experiments. Different letters for the same index indicate significant differences at *p* < 0.05 level.*

### Intercropping Restored the Physiological Activity of Infected Faba Bean Roots Under Cinnamic Acid Stress

**FIGURE 7:**
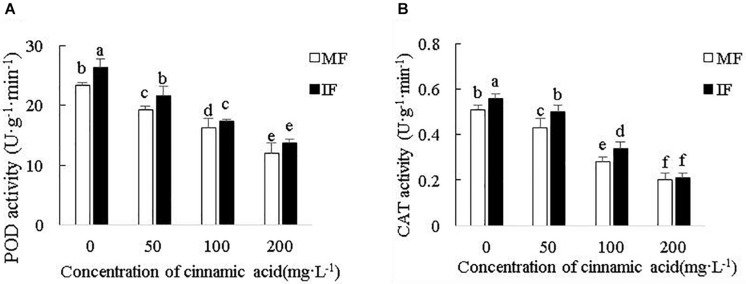
Effect of cinnamic acid on **(A)** POD activity and **(B)** CAT activity in the FOF-inoculated seedlings planted with or without wheat. Data represent mean ± standard error of three biological replicates. Different letters for each index indicate significant differences at *p* < 0.05 level.

Cinnamic acid significantly reduced POD activity in the faba bean roots by 17.15, 30, and 48.56% (Figure 7A), and CAT activity by 15.69, 45.1, and 60.78% (Figure 7B) at 50, 100, and 200 mg/L, respectively, compared with the control. Wheat intercropping increased POD activity relative to the monocrops by 12.86, 12.11, and 6.12% in the presence of 50, 100, and 200 mg/L cinnamic acid, respectively (Figure 7A), and that of CAT activity by 9.8, 16.28, and 21.43% (Figure 7B). However, the increase in enzyme activity was not significant under high dose (200 mg/L) cinnamic acid. Taken together, compared with monocropping under the stress of cinnamic acid, intercropping can effectively alleviate the negative effects of cinnamic acid and increase the activity of antioxidant enzymes of faba beans.

### Intercropping Alleviated Cinnamic Acid-Induced Lipid Peroxidation in Faba Bean Roots

**FIGURE 8:**
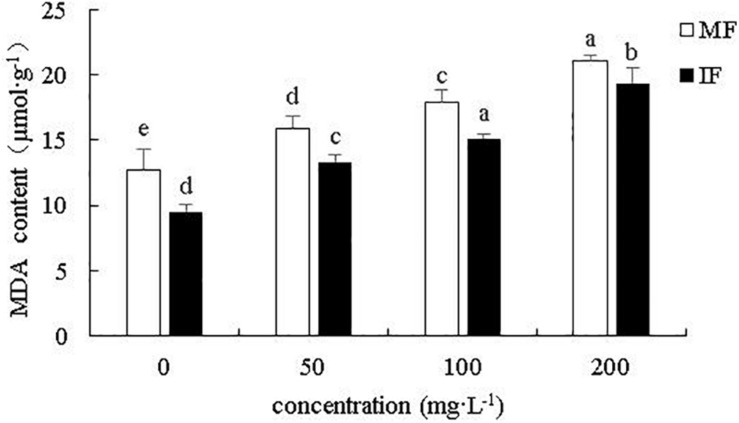
Effect of cinnamic acid on MDA content of monocropped and intercropped faba bean roots after FOF inoculation. Data represent mean ± standard error of three biological replicates. Different letters for each index indicate significant differences at *p* < 0.05 level.

As shown in Figure 8, cinnamic acid significantly increased the content of MDA in monocropped faba bean roots by 24.5, 40.53, and 64.84% at 50, 100, and 200 mg/L, respectively. Wheat intercropping decreased the MDA content relative to monocultured plants by 25.42, 16.33, 15.85, and 8.12%, respectively, under 0, 50, 100, and 200 mg/L cinnamic acid respectively, indicating that intercropping effectively reduces the degree of peroxidation of faba bean roots. Thus, cultivating faba beans with wheat can alleviate cinnamic acid-induced oxidative stress in the former.

### Intercropping Restored Hydrolase Activity in Faba Bean Roots Under Cinnamic Acid Stress

**FIGURE 9:**
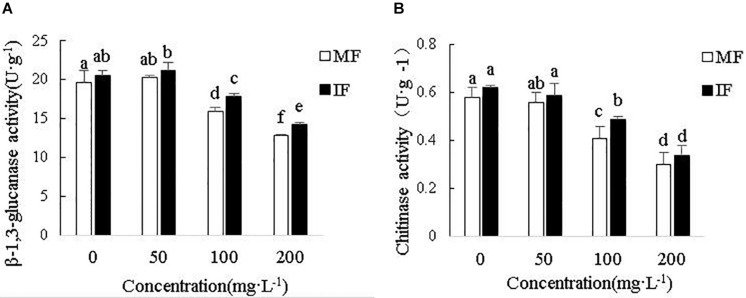
Effect of different concentrations of cinnamic acid on faba bean root **(A)** β-1, 3-glucanase and **(B)** Chitinase activities. Data represent mean ± standard error of three biological replicates. Different letters for each index indicate significant differences at *p* < 0.05 level.

As shown in Figures 9A,B, cinnamic acid inhibited chitinase and β-1, 3-glucanase activities in a concentration-dependent manner, with 200 mg/L showing the maximum inhibition rates of 48.28 and 35.03%, respectively. However, wheat intercropping increased chitinase activity by 19.51 and 13.33%, and that of β-1,3-glucanase by 12.49 and 11.21% in the presence of 100 and 200 mg/L cinnamic acid, respectively. Thus, intercropping effectively alleviated the activity of chitinase and β-1,3-glucanase of faba beans under cinnamic acid stress and indirectly improves the inhibition of pathogens.

## Discussion

Cinnamic acid is an autotoxic substance secreted by faba bean roots that promotes *Fusarium* wilt by directly stimulating pathogenicity of pathogens. Wu et al. (2015) showed that phthalic acid significantly inhibited the growth and sporulation of *Fusarium* but also increased fulvic acid levels and hydrolase activity, which correlate to increased pathogenicity. Consistent with their findings, we found that cinnamic acid inhibited mycelial growth and spore production of *F. oxysporum* but increased cellulase activity by 96.08% and the fusaric acid content by 247.12%. While cellulase destroys the root cell walls and promotes invasion of pathogenic hyphae, the subsequent secretion of fusaric acid destroys the host cell membrane and impairs mitochondrial function and metabolism (Dong et al., 2012). Therefore, we believe that in actual production, when the pathogen is only present in the soil, the damage to the faba bean plant is limited, and the addition of cinnamic acid significantly augments pathogenicity. This will fundamentally increase the occurrence of *Fusarium* wilt.

Root exudates of many crops, such as cucumber, eggplant, and watermelon, contain autotoxic compounds, including erucic acid, syringic acid, vanillic acid (aldehyde), coumaric acid, gallic acid, hydroxybenzoic acid, phthalic acid, and caffeic acid among others that inhibit plant growth and development and facilitate pathogen invasion (Yu et al., 2003; Hao et al., 2006; Chen et al., 2011). For instance, salicylic acid inhibits the growth of patchouli seedlings in a concentration-dependent manner (Xu et al., 2015). Likewise, cinnamic acid enhances the invasion of cucumber roots by *Fusarium*, retarding plant growth by destroying the vascular bundles (Ye et al., 2004). Cinnamic acid and vanillin secreted by eggplant roots also significantly increased the green mud disease index (Chen et al., 2011). Consistent with these studies, we found that cinnamic acid significantly inhibited the growth of faba bean leaves, reduced the plant biomass and height, and increased the incidence and disease index of *Fusarium* wilt in monocultured plants in a concentration-dependent manner. This may be because the increase in the concentration of cinnamic acid significantly promotes pathogen enzyme production, increasing the damage to the faba bean itself and inhibiting the growth of the plants.

Intercropping can obviate the limitations of poor yield and high disease susceptibility seen in continuous single-cropping (Gómez-Rodríguez et al., 2003; Cao et al., 2015). Faba bean and wheat intercropping significantly increased wheat biomass and yield by 19.5–28.2% (Xiao et al., 2018), and intercropping of rice and watermelon significantly increased the yield and height of the latter (Ren et al., 2008). Furthermore, garlic grown with cruciferous vegetables has a lower incidence of white rot (Zewde et al., 2007). Consistent with these findings, wheat and faba bean intercropping not only increased faba bean growth according to various indices but also reduced the incidence and disease index of *Fusarium* wilt under cinnamic acid stress compared to the monocultured plants. In addition, intercropping significantly reduced the content of cinnamic acid in the rhizosphere of faba beans in the field. Therefore after demonstrating that cinnamic acid is an autotoxin secreted by faba beans, we believe that reducing the content of cinnamic acid in the faba bean rhizosphere under field conditions is also an important mechanism for intercropping to alleviate faba bean *Fusarium* wilt.

During adverse conditions, plant cells accumulate free radicals due to reduced antioxidant capacity, leading to oxidative damage of cellular macromolecules and membranes (Yin et al., 2012). Furthermore, autotoxic metabolites produced by the stressed plants accelerate free radical-induced membrane peroxidation and breakdown, thereby providing nutrients to the pathogens and enhancing their ability to invade plant roots. The activity of the antioxidant enzymes POD and CAT are reliable indicators of disease resistance in plants (Ren et al., 2008). Wang et al. showed that exogenous syringic acid and phthalic acid significantly reduced POD and CAT activity in strawberry roots and increased the content of MDA (Wang et al., 2019). Consistent with this, faba bean roots also showed increased oxidative stress when stressed with exogenous cinnamic acid. Intercropping faba beans and wheat significantly enhanced antioxidant enzyme activity in the presence of 50 and 100 mg/L cinnamic acid but was not effective against 200 mg/L. This is likely because the high concentration of cinnamic acid is cytotoxic and the cells are unable to produce CAT and POD in time to relieve the stress. Furthermore, cinnamic acid also increased the content of MDA in the faba bean roots, which correlated with a higher incidence and disease index of wilt and which was alleviated in plants intercropped with wheat. Consistent with our findings, Xu et al. (2015) showed that watermelon and wheat intercropping significantly reduced the MDA content in watermelon roots and improved the antioxidant capacity. Taken together, we speculate that cinnamic acid may increase the occurrence of *Fusarium* wilt by augmenting the oxidative stress of the faba bean root system and inhibiting its ability to relieve stress, while causing further damage to the faba bean root defense system, making pathogen invasion easier. Compared with monocropping, intercropping effectively alleviated the stress of cinnamic acid, improved the antioxidant capacity of faba bean roots, alleviated the oxidative pressure in root cells, and improved the resistance of the faba bean itself. We consider this an important mechanism by which intercropping alleviates faba bean *Fusarium* wilt. Higher plants have evolved elaborate defense systems that are activated in the face of both biotic and abiotic stresses. For instance, plants can resist the invasion of pathogens by releasing defensive substances such as chitinase and β-1, 3-glucanase that can degrade the cell walls of the pathogens. Chitinase hydrolyzes chitin, a major component of fungal cell walls, and prevents hyphal growth, induces rough deformities, and can even disintegrate the cells completely, thus inhibiting pathogenic invasion (Xu et al., 2015). Studies show that ferulic acid inhibited chitinase activity against *F. oxysporum* in a concentration-dependent manner (Ren et al., 2016). The β-1, 3-glucanases hydrolyze β-1, 3-glucans that are present in both bacterial and fungal cell walls decrease the pathogenicity of microorganisms. Xu et al. (2015) found that addition of ferulic acid reduced β-1, 3-glucanase activity in watermelon leaves by 32–37%, and increased disease susceptibility. In this study both β-1, 3 glucanase and chitinase activities decreased in the presence of higher doses of cinnamic acid, further underscoring that cinnamic acid disrupted the faba bean root defense system and aggravated *Fusarium* wilt. In the study by Xu et al. (2015), wheat-watermelon intercropping increased disease resistance in the latter by increasing β-1, 3-glucanase and chitinase activity (Xu et al., 2015). Consistent with this, we found that wheat and faba bean intercropping significantly increased the activity of both enzymes in faba bean roots compared to monocultured plants. We believe that cinnamic acid can create a safer living environment for pathogens by inhibiting the activity of β-1, 3 glucanase and chitinase, which may increase the reproduction density of the pathogens and promote the development of *Fusarium* wilt. Compared with monocropping, intercropping effectively alleviated the inhibitory effect of cinnamic acid on chitinase and β-1, 3-glucanase activity which may lead to a decrease in the pathogen reproduction density and contribute to the alleviation of faba bean fusarium wilt. Therefore, we consider that improving the resistance of the faba bean itself and the activity of resistant substances is an important mechanism for intercropping to effectively alleviate faba bean *Fusarium* wilt.

We simulated changes in the physiological resistance of faba bean roots and the mitigating effects of intercropping under cinnamic acid stress in a hydroponic system, which has higher cinnamic acid concentrations compared to soil due to microbial degradation of phenolic acids in the latter. Li et al. (2014) found that benzoic acid added to unsterilized soil at 170 mg/kg was completely degraded in about 7 days. Thus, the actual content of phenolic acid released from the roots in the soil or residue decay is generally higher than the measured concentration. Therefore, the actual role of cinnamic acid in the continuous single cropping of faba bean needs to be validated by soil culture experiments.

In general, cinnamic acid can increase the occurrence of wilt by stimulating pathogen enzyme production and breaking the defense system of the faba bean root system. Faba bean and wheat intercropping effectively controlled the occurrence of wilt by increasing the defense enzyme activity and alleviated the self-toxic effects of cinnamic acid. It cannot be ignored that the intercropping significantly reduces the content of cinnamic acid in the rhizosphere by alleviating the stress of cinnamic acid.

Using biodiversity to control plant diseases has become a hot spot in domestic and foreign research in recent years (Wolfe, 2000). In this study, cinnamic acid increased the occurrence of faba bean *Fusarium* wilt by destroying the defense system of the faba bean root system and stimulating FOF to produce mycotoxins and pathogenic enzymes. As an agricultural method to control diseases, intercropping not only significantly reduces the amount of cinnamic acid but also effectively alleviates the autotoxicity of cinnamic acid and controls the occurrence of *Fusarium* wilt by improving the activity of defensive enzymes. Our previous research also found that, under field conditions, wheat/faba bean intercropping controlled faba bean *Fusarium* wilt and increased the faba bean yield (Hu et al., 2016; Guo et al., 2020). However, it cannot be ignored that in actual field production, the effect of intercropping to control disease is often the result of multiple factors. For example, different fertility conditions, soil types, climate factors, and even different intercropping ratios may have an impact. This is interesting and worthy of further study. In addition, the effect of intercropping on disease control is also limited, just as the intercropping in this study cannot alleviate the toxicity caused by 200 mg/ml cinnamic acid. The reason may be that under such strong dual stresses, the cells are severely damaged and the normal physiological and biochemical functions are lost. Furthermore, the cellular responses and molecular events that occur during the plant-phenolic acid-*Fusarium* interaction in the faba bean/wheat intercropping system may also provide important insights for the control of faba bean *Fusarium* wilt by intercropping, requiring further study and exploration of its mechanism.

## Data Availability Statement

The original contributions presented in the study are included in the article/supplementary material, further inquiries can be directed to the corresponding author/s.

## Author Contributions

YG conceived the original screening and research plans, finished writing this thesis. YD and KD supervised the experiments, agreed to serve as the author responsible for contact and ensures communication. JL provided technical assistance to YG. YG designed the experiments. QZ analyzed the data. All authors contributed to the article and approved the submitted version.

## Conflict of Interest

The authors declare that the research was conducted in the absence of any commercial or financial relationships that could be construed as a potential conflict of interest.

## Abbreviations

FOF: *Fusarium oxysporum*
POD: Peroxidase
CAT: Catalase
MDA: Malondialdehyde
SOD: Superoxide dismutase
GPX: Glutathione Peroxidase
APX: Ascorbate peroxidase
MF: Monocropped
IF: Faba bean intercropped with wheat
FON: Fusarium oxysporum f.sp.niveum.
